# Knowledge and attitude on prevention of COVID-19 among community health workers in Nepal-a cross-sectional study

**DOI:** 10.1186/s12889-021-11400-9

**Published:** 2021-07-19

**Authors:** Amul Shrestha, Tek Bahadur Thapa, Mahendra Giri, Sanjiv Kumar, Sakil Dhobi, Haikali Thapa, Pawan Prakash Dhami, Arun Shahi, Ajita Ghimire, Ela Singh Rathaur

**Affiliations:** 1 IFHR (Innovative Foundation for Health and Research), Kathmandu, Nepal; 2 Department of Public Health, Health Office, Lalitpur, Bagmati Province Nepal; 3 Dhulikhel Hospital, Kathmandu University Hospital, Dhulikhel, Nepal; 4 Department of Social Welfare Health Section, Lalitpur Metropolitan City, Pulchowk, Lalitpur, Nepal; 5 Department of Public Health, Tarkeshwor Municipality, Kathmandu Nepal; 6 Consultant of medical oncology, Patan Academy of Health Sciences, Lalitpur, Nepal; 7 Consultant-Disease Surveillance for emergency response- Strengthening System for Better Health, Lalitpur, Nepal

**Keywords:** Knowledge and attitude, Community health care workers (HCW’s), Enabling factors, Palika (rural/ urban municipalities and metropolitan city)

## Abstract

**Background:**

Emerging and reemerging pathogens are global challenges for public health and the pandemic of Coronavirus disease 2019is a reemerging case of an infectious disease caused by Severe Acute Respiratory Syndrome-Corornavirus-2. Health care worker worldwide are at higher risk worldwide and the situation is the same in Nepal. The knowledge and attitude of health workers will certainly mark the outcome towards this pandemic. So, this study aims to assess the knowledge and attitude of community health workers towards the prevention of COVID-19 virus.

**Methods:**

A descriptive cross-sectional study was carried out among the community health workers of various provinces of Nepal. A semi-structured, self-administered questionnaire was prepared in Google form and circulated to the sampled health workers through various social media platforms like face book, messenger, Instagram and g-mails. A total of 650 invitations were send and among them 420 responded and among them only 399 provided complete response. Responses containing anonymized data was collected analyzed in using SPSS-version-20. The results were interpreted and was checked with various demographic and enabling factors using chi-square test and logistic regression model. Also, ethical approval was taken from NHRC (Nepal Health Research Council (protocol registration number: 360/2020P) prior to the conduction of study.

**Results:**

Out the total sample size of 450, we took 399 responses taking into consideration all the inclusion criteria. So, from 399 valid response, 230 (47.6%) were males and 169 (42.4%) were females. 380 (95.2%) employed participants thought that wearing PPE will reduce the chances of getting COVID-19, majority of the participants 80.5% (321) responded that COVID-19 will successfully be controlled and staffs receiving excellent support from palika had high knowledge level. Knowledge level was significantly associated with the enabling factor “support from palika” (*P* = 0.045).45.9% of the respondents had no availability of COVID-19 response medical items for prevention of COVID-19 at their respective health facilities. Also the logistic regression analysis revealed that the odds of knowledge level was 2 times higher (AOR=1.913 at 95% CI: 1.266-2.891) compared to the female participants (Ref- female).

**Conclusions:**

Proper and adequate knowledge and attitude towards COVID-19 is a paramount in the prevention and control of SARS-COV-2. Health care workers are knowledgeable about COVID-19 and are proactively practicing the preventive measures to minimize the spread of infection but some lack optimistic attitude. Hence, the constantly updated educational programs related to COVID-19 will surely contribute to improving the healthcare workers knowledge and attitude towards COVID-19.

**Supplementary Information:**

The online version contains supplementary material available at 10.1186/s12889-021-11400-9.

## Background

Emerging and reemerging pathogens are global challenges for public health [1]. Corona viruses are enveloped RNA viruses that are distributed broadly among humans, other mammals, and birds and causes respiratory, enteric, hepatic, and neurologic diseases [2, 3]. Six coronavirus species are known to cause human disease [4]. Four viruses 229E, OC43, NL63, and HKU1 are prevalent and typically cause common cold symptoms in immunocompromised individuals [4]. The two other strains, severe acute respiratory syndrome coronavirus (SARS-CoV) and Middle East respiratory syndrome (MERS) coronavirus (MERS-CoV) are zoonotic in origin and have been linked to sometimes fatal illness [5]. Corona virus disease 2019 (COVID-19) is an infectious respiratory disease which is caused by newly recognized corona virus [5–7]. Given the high prevalence and wide distribution of corona viruses, the large genetic diversity and frequent recombination of their genomes, and increasing human–animal interface activities, novel corona viruses are likely to emerge periodically in humans owing to frequent cross-species infections and occasional spillover events [5, 8, 9].

In late December 2019, several local health facilities reported clusters of patients with pneumonia of unknown cause that were epidemiologically linked to a seafood and wet animal wholesale market in Wuhan, Hubei Province, China [10]. In January 2020, the outbreak of the 2019 novel coronavirus (2019-nCoV) in China spread progressively to other countries [11, 12], with WHO declaring it a Public Health Emergency of International Concern [13]. Among the affected 200 countries globally (where 2,074,529 confirm cases and 139,378 deaths were reported as of April; and 23,560 confirm cases and 586 deaths South East Asia region including Nepal [14]. On 23rd January 2020, the first case was reported for COVID-19 in Nepal and now (21st Sept 2020), the data hiked at 64,122 cases with 46,233 recovered and 411 deaths [15].

Health workers are at the front line of the COVID-19 outbreak response and as such are exposed to hazards that put them at risk of infection [16]. Community health workers are one of the apex part of the health system of Nepal. After the decentralization, local government health system purely depends on community health workers. They are the pioneer for the prevention, promotion, and curative activities at the community level. So, their knowledge and attitude play a pivotal role for preventing COVID-19 at the community level and this study aims to find out the knowledge and attitude on prevention of novel coronavirus among community health workers in Nepal.

## Methods

A descriptive cross-sectional study design was carried out to find out the status of knowledge and attitude of community health care workers towards the prevention of novel corona virus-2019. The study was conducted among the community health workers who are working at Health post and Primary Health Care level health institutions during the pandemic of COVID-19 in all the seven provinces of Nepal.

A semi-structured, self-administered questionnaire was prepared in google form and circulated to the sampled health workers of all 7 provinces. The online forms were disseminated via various social media platforms like Facebook, messenger, Instagram, g-mails, Whatsaap and Viber. The informed consent was also attached along with the Google forms before the beginning of the survey. Informed consent was taken from each respondent before the data collection and they were provided full rights to withdraw the study at any instance of time they wish. Also, ethical approval was taken from NHRC (ERB (Ethical Review Board) protocol registration number: 360/2020 P) prior to the conduction of study. A total of 650 invitation were send and among them 420 responded and among them only 399 provided complete response.

The sample size was calculated using the formula for the descriptive cross-sectional study, i.e. *N* = (Z _α/2)_^2^ p*q / d^2^) where, Z _α/2=_ Z-normal score corresponding to 95% CI i.e. 1.96*p* = prevalence of knowledge on COVID-19 = 75%, q = 1-p and d = allowable error = 4%. The sample size *N* = {(1.96) ^2 *0.75* 0.25/ (0.04)^2} = 450.The sampling of the respondents was carried out via convenience sampling technique (non-probability sampling method). For maintaining quality control and quality assurance of this study, designed questionnaires was pre-tested for content validity and tools were modified after pre-test and from observation field note. After the distribution of the questionnaire, 420 responses were obtained from the health care workers out of which 399 met the inclusion criteria which was 95% of the total response and 88% of the total sample size.

After the data collection, it was checked for completeness. The information will automatically be transferred to excel sheet and then transferred to SPSS. Responses containing anonymized data was collected and analyzed using SPSS-version 20. The results were interpreted in terms of percentage response, knowledge and attitude score and chi-square test and logistic regression was adopted to find the association between knowledge and attitude with various demographic and enabling factors.

## Results

Out the total sample size of 450, we took 399 responses taking into consideration all the inclusion criteria. Responses from health workers other than HP, PHC of Government of Nepal, not residing in Nepal, not working at the time of COVID-19 pandemic, were excluded. So, from 399 valid response, 230 (47.6%) were males and 169 (42.4%) were females. So, the gender of the participants was almost evenly distributed. Regarding the marital status of the respondents, more than half of them were married (61.7%) followed by unmarried (37.6%). Among the total respondents participated, nearly 1/3rd was HA (Health Assistant) (29.6%) and the least were ANM and staff nurses, both 4.8%. Majority of the respondent (61.2%) said that Television and radio was the media through which they firstly got the information about COVID-19. Nearly 2/5 (40.1%) of the respondents were working at Bagmati province while the least (3%) respondents were from Sudurpaschim province. Regarding the education level, 41.4% of the respondents had completed PCL (Proficiency Certificate level) and only 11.3% had completed their master level study. Nearly half (46.1%) of the respondents responded that their monthly income was between 25 k–35 k which was the average scale of government employee of Nepal of 5/6th level. The information is showed in Table 1.

**Table 1:** Socio-demographic characteristics of participants (*n* = 399)

Variables	Category	Frequency	Percentage
Age	18–27	177	44.4
	28–37	137	34.3
	38–47	63	15.8
	48 and above	22	5.5
		**399**	**100.0**
Gender	Male	230	57.6
	Female	169	42.4
		**399**	**100**
Marital status	Married	246	61.7
	Unmarried	150	37.6
	Divorced	1	0.3
	Widow	2	0.5
			**100**
Designation	HA	118	29.6
	AHW	87	21.8
	ANM	97	24.3
	Nursing staff	19	4.8
	PHO	19	4.8
	Others	59	14.8
		**399**	**100**
Firstly, known Information about COVID-19	Socialmedias (FB/Twitter/ YouTube/ Instagram)	137	34.3
	Television/ radio	244	61.2
	Friends and relatives	7	1.8
	scientific journals	11	2.8
		**399**	**100**
Working province	Province 2	123	30.8
	Bagmati	160	40.1
	Gandaki	6	1.5
	Karnali	98	24.6
	Sudurpaschim	12	3.0
		**399**	**100**
Education level	Master	45	11.3
	Bachelor	102	25.6
	PCL	165	41.4
	TSLC	87	21.8
		**399**	**100**
Monthly income	25 K–35 K	184	46.1
	35 K–45 K	92	23.1
	45 K–55 K	62	15.5
	Above 55 K	61	15.3
		**399**	**100**
Orientation on COVID-19	Yes	82	20.6
	No	317	79.4
		**399**	**100**
Extra allowance provided	Yes	94	23.6
	No	305	76.4
		**399**	**100**
Working hours in a week	<=40	119	29.8
	> 40	280	70.2
		**399**	**100**
Support from palika	Excellent	17	4.3
	Good	251	62.9
	Poor	79	19.8
	very Poor	52	13.0
		**399**	**100**
Palika level COVID-19 Response team	Yes	281	70.4
	No	23	5.8
	Don’t know	95	23.8
		**399**	**100**

Table 1: Frequency regarding the various enabling factors had different responses. It was found from the study that very minimum (20.6%) employee has received orientation on COVID-19 and also very few staffs (23.6%) received extra allowance during the situation of COVID-19 and the rest did not get any allowance which might demotivated them in their performance. Regarding the working hours of health staffs, more than 2/3 (70.2%) said that they had been working for more than 40 h in a week due to the situation of COVID-19. Support from respective palikas was found good (62.9%) which might have motivate them to work efficiently; maximum respondents (70.4%) said that they had “palika level COVID-19 response team” for the surveillance of situation of COVID-19.

In the knowledge related section shown in Table 2, almost all, 99.5% (397) participants said that COVID-19 is caused by virus and maximum, 98.5% (393) responded that COVID-19 is transmitted by close contact with infected people. Regarding the knowledge about the first case of novel corona virus identified place, it was found that majority of the participants, 90.2% (360) know that it was found firstly in Wuhan, China. Furthermore, regarding the knowledge about symptoms of COVID-19, 87.2% (348) said that fever, cough, shortness of breath was the symptoms of corona virus. Regarding the isolation period of COVID-19, majority of the participants, 70.7% (282) were well known about it and 82.2% (328) had right knowledge about prevention from COVID-19. Remarkably, all 100% of the respondents were well known about the fact that antibiotic was not the first line treatment of COVID-19. A total of 88.3% (352) participants correctly said that PPE includes gloves, apron, face mask and eye protection.

**Table 2:** Frequency related to knowledge of participants

Variables	Category	Frequency	Percentage
COVID-19 is caused by	Virus	397	99.5
	Fungus	1	0.3
	Parasite	1	0.3
		**399**	**100**
COVID-19 is transmitted by close contact with infected person?	Yes	393	98.5
	No	6	1.5
		**399**	**100**
The first case of novel coronavirus was identified in	Beijing	17	4.3
	Shanghai	22	5.5
	Wuhan, Hubei	360	90.2
		**399**	**100**
Which is true about COVID-19?	All age group	87	21.8
	Mild in children	9	2.3
	Older and pre-existing medical condition	94	23.6
	All of the above	209	52.4
		**399**	**100**
Symptoms of COVID-19 are:	Fever	7	1.8
	Cough	3	0.8
	Shortness of breadth	41	10.3
	All the above	348	87.2
		**399**	**100**
The COVID-19 virus spreads via respiratory droplets of infected individuals	Yes	395	99
	No	4	1
		**399**	**100**
The isolation period for COVID-19 is:	1 week	3	0.8
	2 weeks	282	70.7
	3 weeks	74	18.5
	More than 4 weeks	40	10
		**399**	**100**
How to prevent from COVID19?	Clean hands with soap and water/ sanitizer	57	14.3
	Cover mouth and nose when coughing and sneezing	6	1.5
	Avoid close contact with those who show signs of flu	5	1.3
	Properly cook meats, eggs before eating	3	0.8
	All the above	328	82.2
		**399**	**100**
COVID-19 vaccines are available in the market now	Yes	54	13.5
	No	345	86.5
		**399**	**100**
Health care workers are at high risk of COVID-19	Yes	389	97.5
	No	10	2.5
		**399**	**100**
PPE includes:	Gloves	16	4
	Gloves, apron	20	5
	Gloves, apron, face mask	11	2.7
	Gloves, apron, face mask and eye protection	352	88.3
		**399**	**100**

Regarding the attitude of the respondents, 56% (224) had the feeling that they will get infected with COVID-19 at some time of the pandemic period, 92% (367) accepted that they will be in isolation in health facility if they are infected with COVID-19. Only 11.5% (46) participants refuse to take the vaccine if it was available in the market. Furthermore, 95.2% (380) employed participants thought that wearing PPE will reduce the chances of getting COVID-19. Also, regarding the COVID − 19 prevention, 92.5% (369) participants thought that lockdown was the best way. Regarding the attitude and perception towards the control of COVID-19, majority of the participants 80.5% (321) responded that COVID-19 will successfully be controlled followed by 19% (78) who thought that it won’t be controlled at the earliest. This is illustrated in Table 3.

**Table 3:** Frequency related to attitude variables of participants

Variables	Categories	Frequency	Percentage
Do you think you will get ill from COVID-19	Yes	224	56.1
	No	175	43.9
		**399**	**100**
If you get COVID-19, will you accept isolation in a Health facility?	Yes	367	92
	No	32	8
		**399**	**100**
If COVID-19 vaccine were available, would you t take it?	Yes	353	88.5
	No	46	11.5
		**399**	**100**
Do you think wearing PPE reduces the chance of getting COVID-19?	Yes	380	95.2
	No	19	4.8
		**399**	**100**
Do you think lockdown is the best way to prevent COVID-19?	Yes	369	92.5
	No	30	7.5
		**399**	**100**
Do you feel bad when people don’t use masks while coming for treatment?	Yes	367	92
	No	32	8
		**399**	**100**
Do you think COVID-19 will successfully be controlled?	Yes	321	80.5
	No	78	19.5
		**399**	**100**

In bivariate analysis (chi-square test) Table 4, it was found that gender was significantly associated with knowledge level as male tend to have more knowledge level (*P* < 0.002). Also, marital status was found to be significantly associated with the knowledge scores, for married knowledge level found to be high (*P* = 0.008) compared to the other categories. It was also found that participants working at Bagmati province had high level of knowledge (*P* < 0.001). Participants who were designated as “HA” and education level- PLC complete was significantly associated with the knowledge level. Similarly, people with monthly income 25 k–35 k was found to have more knowledge level (*P* < 0.005) and also the significant association with knowledge level was found with the enabling factor “support from palika” (*P* < 0.045). Staffs receiving excellent support from palika had high knowledge level.

**Table 4:** Association of Knowledge with demographic and enabling factors

Variables	Categories	Knowledge		***P***-value
		**High**	**Low**	
Age	18–27	113	64	0.924
	28–37	88	49	
	38–47	28	25	
	48 and above	13	9	
Gender	Male	160	70	***0.002***
	Female	92	77	
Marital status	Married	142	104	***0.008***
	Unmarried	108	42	
	Divorced	1	0	
	Widow	1	1	
Designation	HA	81	37	***< 0.001***
	AHW	56	31	
	ANM	41	56	
	Nursing staff	17	2	
	PHO	16	3	
	Others	41	18	
Firstly, known Information about COVID-19	Social medias (FB/Twitter/ youtube/ instagram)	101	36	***< 0.001***
	Television/ radio	134	110	
	Friends and relatives	6	1	
	scientific journals	11	0	
working province	Province 2	86	37	***< 0.001***
	Bagmati	111	49	
	Gandaki	5	1	
	Karnali	45	53	
	Sudurpaschim	5	7	
Education level	Master	29	16	***< 0.001***
	Bachelor	68	34	
	PCL	121	44	
	TSLC	34	53	
Monthly income	25 K–35 K	118	66	***0.005***
	35 K–45 K	45	47	
	45 K–55 K	44	18	
	Above 55 K	45	16	
Orientation on COVID-19	Yes	50	32	0.646
	No	202	115	
Working hours in a week	<=40	74	45	0.793
	> 40	178	102	
Extra allowance provided	Yes	57	37	0.562
	No	195	110	
Support from palika	Excellent	10	7	***0.045***
	Good	153	98	
	Poor	60	19	
	very Poor	29	23	
Sufficient equipment and medicines available at health facilities	Yes	131	121	0.259
	No	85	62	
Palika level COVID-19 Response team	Yes	179	102	0.879
	No	15	8	
	Don’t know	58	37	

On the other hand, gender, marital status, working province and presence/ absence of palika level COVID-19 response team had significant association with the attitude score. Male participants (*P* < 0.001), HA (*P* < 0.001), staffs working at Bagmati province (*P* < 0.01) and participants working in palika having “palika level COVID-19 response team” (*P* < 0.001) was found to have positive attitude toward the prevention of COVID-19 compared to others Table 5.

**Table 5:** Association with Attitude and demographic/ enabling factors

Variables	Categories	Attitude		*P*-value
		**Positive**	**Negative**	
Age	18–27	123	54	0.26
	28–37	84	53	
	38–47	39	24	
	48 and above	13	5	
Gender	Male	167	63	***< 0.001***
	Female	96	73	
Marital status	Married	154	92	0.12
	Unmarried	108	42	
	Divorced	0	1	
	Widow	1	1	
Designation	HA	90	28	***< 0.001***
	AHW	56	31	
	ANM	49	48	
	Nursing staff	11	8	
	PHO	16	3	
	Others	41	18	
Firstly, known Information about COVID-19	Social medias (FB/Twitter/ YouTube/ Instagram)	104	33	***0.002***
	Television/ radio	144	100	
	Friends and relatives	6	1	
	scientific journals	9	2	
working province	Province 2	86	37	***0.01***
	Bagmati	114	46	
	Gandaki	5	1	
	Karnali	50	48	
	Sudurpaschim	8	4	
Education level	Master	31	14	0.09
	Bachelor	67	35	
	PCL	117	48	
	TSLC	48	39	
Monthly income	25 K–35 K	128	56	0.184
	35 K–45 K	52	40	
	45 K–55 K	42	20	
	Above 55 K	41	20	
Orientation on COVID-19	Yes	54	28	0.99
	No	209	108	
Working hours in a week	<=40	82	37	0.411
	> 40	181	99	
Extra allowance provided	Yes	60	34	0.626
	No	203	102	
Support from palika	Excellent	11	6	0.309
	Good	164	87	
	Poor	58	21	
	very Poor	30	22	
Sufficient equipment and medicines available at health facilities	Yes	123	60	0.615
	No	140	76	
Palika level COVID-19 Response team	Yes	191	90	***0.001***
	No	21	2	
	Don’t know	51	44	

### Stepwise logistic regression

We performed forward stepwise logistic regression to identify whether the demographic and enabling factors had significant association with knowledge and attitude status of the participants. In Table 6, the logistic regression analysis revealed that the odds of knowledge level was 2 times higher (AOR = 1.913 at 95% CI: 1.266–2.891) compared to the female participants. Knowledge level was significantly associated with the designation of the respondents as nursing staffs had 2 folds more knowledge (AOR = 2.243, 95% CI = 1.006–5.00) compared to those of the HA (Ref-HA). Similarly, the variables education level and monthly income of the participants were also found to have statistically significant relationship with the knowledge level.

**Table 6:** Effect of socio-demographic and enabling factors on Knowledge and attitude (logistic regression model)

**Variables**	**Categories**	**Adjusted Odds ratio (AOR)**	**95% CI**	***P-value***
Gender	Male	1.913	1.266–2.891	***0.002***
	Female	Ref		
Designation	HA	Ref		
	AHW	1.213	0.563–2.613	0.622
	ANM	0.991	0.450–2.181	0.982
	Nursing staff	2.243	1.006–5.000	***0.048***
	PHO	0.279	0.055–1.429	0.126
	Others	0.386	0.092–1.620	0.193
Education level	Master	Ref		
	Bachelor	0.465	0.228–0.950	***0.036***
	PCL	0.778	0.311–1.949	0.592
	TSLC	0.305	0.161–0.579	***< 0.001***
Monthly income	25 K–35 K	Ref		
	35 K–45 K	1.338	0.647–2.766	0.431
	45 K–55 K	2.523	1.157–5.500	***0.020***
	Above 55 K	0.979	0.415–2.312	0.962
**Variable**	**Categories**	**Adjusted Odds ratio (AOR)**	**95% CI**	***p-value***
Gender	Male	1.884	1.216–2.918	***0.005***
	Female	Ref		
Firstly, known Information about COVID-19	Social medias (FB/Twitter/ YouTube/ Instagram)	Ref		
	Television/ radio	0.301	0.035–2.580	0.273
	Friends and relatives	0.367	0.076–1.768	0.211
	scientific journals	0.475	0.293–0.768	***0.002***
Palika level COVID-19 Response team	Yes	Ref		
	No	1.451	0.882–2.386	0.142
	Do not know	0.195	0.044–0.863	***0.031***

Furthermore, attitude status of the respondents was also found to have significant association with the variables like gender, firstly known information about COVID-19 and presence/ absence of palika level COVID-19 response team. Here, it was found that male tend to have positive attitude which is 2 times more (AOR: 1.884, 95% CI: 1.216–2.918) compared to those of the females (ref-female). The other two variable are shown in the Table 6.

## Discussion

The study investigated the knowledge and attitude of community health care providers who were working during the time of COVID-19 pandemic at health facilities of Nepal. The result showed that gender, marital status, designation, information about COVID-19, working province, education level, monthly income, support from palika were found statistically significant with knowledge level and attitude status was associated with gender, designation, information about COVID-19, working province and palika level COVID-19 response team. HCWs are the frontline workers in the management of suspected and potential cases of the COVID-19. Their knowledge and attitude will likely have an important bearing on the course and containment of the pandemic.

Our findings revealed that most of the participants (63.2%) were knowledgeable about the prevention of COVID-19. The finding is consistent with other studies done at china, Indian and Saudi Arabia [17–20]. The high level of knowledge might be due to the information network of the current modern world and higher education level of the people. On the other hand, the attitude was also found positive among more than 2/3rd (65.9%) of the participants which was also correlated with various studies conducted at China India, Vietnam and Saudi Arabia [17–20]. The gender of the respondents was much evenly distributed as 47.6% male and 42.4% females which we considered a good gender response rate. Nearly half (46.1%) of the respondents responded that their monthly income was between 25 k–35 k which was the average scale of government employee of Nepal of 5/6th level and our almost respondents fall on this category. Regarding the enabling factors, it was found that very less (20.6%) participants had received orientation on COVID-19 but maximum (70.4%) said that they had palika level COVID-19 response team for the surveillance of the situation of COVID-19.

Regarding the knowledge level, nearly all the participants (98.5%) know that COVID-19 is transmitted by close contact with the infected people and 90% were correct regarding the place of outbreak of Corona virus i.e. Wuhan, China. Furthermore, regarding the knowledge about symptoms of COVID-19, 87.2% (348) said that fever, cough, shortness of breath was the symptoms of corona virus which was a very good response rate. The high rate might be because of the field and area they were working i.e. medical field. Regarding the isolation period of COVID-19, majority of the participants, 70.7% (282) were well known about it and 82.2% (328) had correct knowledge about prevention from COVID-19. This finding was consistent with a recent study done in Nepal [21]. Remarkably, all 100% of the respondents were well known about the fact that antibiotic was not the first line treatment of COVID-19.

Concerning the attitude of the respondents, more than half of the respondents (56%) had the feeling that they will be infected with Corona virus at some point of time of the pandemic. The positive point is that majority of the respondents (92%) accepted that they will stay at isolation if they get infected with corona virus. This is a very positive attitude of the respondents and especially the health workers. On the other hand, still 8% did not accept it, which is likely to be related to a lack of knowledge within the HCW’s about current and important prevention and isolation strategies. A study from Taiwan also exhibit a similar result [22]. Majority of the respondents (80.5%) were positive that COVID-19 will successfully be controlled which is a positive attitude toward any health problems. Positive attitudes and high confidence in the control of COVID-19 can be explained by the government’s unprecedented actions and prompt response in taking stringent control and precautionary measures against COVID-19, to safeguard citizens and ensure their well-being. These measures include the lockdown, and the suspension of all domestic and international flights, schools and universities, and the stepwise shutdown and prohibitory orders imposed.

In the study it was found that gender was associated with both knowledge level and attitude of the respondents. Male were found to have more knowledge and positive attitude toward the prevention of COVID-19 (*p* < 0.002). This was contrary to the finding of the study conducted at India and China [23, 24]. The reason for male to be more in this category might be that in Nepal, still male get higher chance to get exposed in various field and orientation programs. Females are not highly entertained in the training and seminars conducted because of the thinking that they have had the responsibilities at their home and couldn’t contribute fully for the programs.

Television and radio were significantly associated with the knowledge and attitude of the respondents as higher level of knowledge was associated with those who get information via TV and radio. This might bedue to the reason that TV and radio are the reliable Medias [25] and only broadcast the information based on evidences despite some of the social medias like Facebook, You-tube, Instagrametc [26, 27]. Also, education level (< 0.001), working province (p < 0.001) and monthly income (*p* < 0.005) are some of the demographic characteristics which have association with the knowledge level and regarding the enabling factors only “support from palika” is found to be associated with the knowledge level (*p* < 0.045). Since this, study assessed only limited demographic variables, it is recommended that we include more demographic as well as socio-cultural variables in further studies.

This study also highlighted the area of some enabling factors which might be responsible for the knowledge and attitude of the HCW’s. Most of the respondents (79.4%) had not received any orientation regarding COVID-19. This might have serious impact on the knowledge level of the HCW’s [17, 28]. Another finding of this study revealed that 70% of the respondents work for more than 40 h in a week and nearly 3/5th (76.4%) is not paid extra allowance during their extra hours of services. So, this will really demotivate the HCW’s toward their dedicated services. This issue should be addressed by the concerned authority as soon as possible. Many of the palikas, HCW’s work have a palika level COVID-19 response team (70.4%) followed by 5.8% not having and 23.8% were not aware of it. HCW’s not being aware of the palika level COVID-19 response team clarifies that some of them are still not serious and concerned about their roles and responsibilities.

Nevertheless, the study findings revealed that the knowledge and attitude of the respondents/ HCW’s working at various health facilities at different provinces of Nepal was found to be good despite some had low level of knowledge and negative attitude toward the prevention of COVID-19. The logistic regression (forward stepwise) also revealed several factors being associated with the knowledge and attitude of the HCW’s. Among then some were found matching with various studies conducted at national/ international level [17, 28–30] whereas some were found to contradict with the various study findings [21–24].

Our study also has some notable limitations. First, as those who had no internet access could not take part in the survey as the questionnaire were circulated through google forms. Also, the study could not take many variables of prime focus into consideration due to shorten the length of the questionnaire as it was self-administered and online based. Furthermore, since the attitudes are based on the healthcare workers’ knowledge and availability of specialized logistics like PPE for maintaining the appropriate biosafety along with their perception of the healthcare system, their understanding of institutional preparedness are solely based on their own observation and perception.

Finally, the study aims to explore the ground reality of the knowledge and attitude of HCW’s toward the prevention from COVID-19 in Nepal. This is an utmost need in the context of this pandemic to diminish the situation from worsening further. This study will eventually bridge the gap between the current situation of COVID-19 of the HCW’s and the policy makers at the local, province and federal level of Government of Nepal. There is a need to educate and orient the HCW’s regarding COVID-19 via different method and medias appropriate at the current situation of pandemic to upgrade the knowledge level of the HCW’s and also building the HCW’s confidence on existing health system regarding appropriate and timely containment of the pandemic so improving the trust and reliability between government policy makers and the HCW’s is of paramount importance.

The results showed that healthcare workers report good knowledge and practices related to COVID-19. However, they lack optimistic attitudes and confidence. The findings also demonstrated that healthcare professionals seeking formation from unverified sources such as social media and co-workers. These results are impactful and should be addressed through standardized training opportunities and distribution of official sources about COVID-19 to health care professionals to deliver optimal care to COVID patients and to minimize the risk of transmission of infection among health workers. Constantly updated refresher training from authentic sources will contribute to better performance.

## Conclusion

Proper and adequate knowledge and attitude towards COVID-19 is a paramount in the prevention and control of SARS-COV2 and it is utmost important to the frontline health workers who are daily exposed to the risk of virus. Knowing the causes and transmission sources of a disease, increases the likelihood that people will become more aware of the spread of communicable diseases, and of the preventive measures to slow the transmission. Although most of the study participants possess a good knowledge and positive attitude toward the prevention of COVID-19, still there is a need to orient more HCW’s regarding COVID-19preventive measures. These results are impactful and should be addressed through standardized training opportunities and distribution of official sources about COVID-19 to healthcare professionals to deliver optimal care to COVID patients and to minimize the risk of transmission of infection among health workers. Constantly updated refresher training from authentic sources will contribute to better performance.

## Supplementary Information

**Additional file 1:** Questionnaires.

**Additional file 2:** COVID-19 database SPSS.

## Data Availability

All data generated or analyzed during the study is attached in the additional supporting files. The datasets used and analyzed for the study is also available from the corresponding author on reasonable request. The e-mail id of the corresponding author is: *shresthamul98@gmail.com*

## Abbreviations

COVID-19: Corona Virus Disease-19
SARS: Severe Acute Respiratory Syndrome
HCW’s: Health Care Workers
PPE: Personal Protective equipment
PCL: Proficiency Certificate Level
TSLC: Technical School Leaving Certificate
HP: Health Post
PHCC: Primary Health Care Center
GoN: Government of Nepal
