# Supplementary material for: Knowledge and attitude on prevention of COVID-19 among community health workers in Nepal-a cross-sectional study
Source: BMC Public Health. 2021 Jul 19;21:1424. doi: 10.1186/s12889-021-11400-9 (PMC8287280; doi:10.1186/s12889-021-11400-9)
Supplement: Supplementary file 1 — Additional file 1: Questionnaires. [file 12889_2021_11400_MOESM1_ESM.docx]

**ANNEX - I**

**Questionnaires**

**Section A: Demographic Information**

 1.      Name: ……………………

 2.  Age:

| a. 18-27 |
| --- |
| b. 28-37 |
| c. 38-47 |
| d. 48 and above |
|  |

 3        Sex: a. M b. F  c. Other

 4.     Marital status:   a. married     b. unmarried            c. divorced

 5.      How did you know about COVID-19?

a.   Television

b.  Social media (Facebook, twitter, YouTube)/ internet

c.   Friends/ relatives

d.  Scientific articles / journals

e.   Do not know

 6.      Which province do you work?

a.   Province no. 1

b.  Province no.2

c.   Bagmati Province

d.  Gandaki Province

e.   Province no.5

f.        Karnali Province

g.  Sudurpaschim Province

| 7.      Designation:  a. HA |
| --- |
| b. AHW |
| c. ANM |
| d. Nursing staff |
| e. PHO |
| f. Others |

 8.      Education Level (Health Sciences)

a.   TSLC

b.  PCL

c.   Bachelor

d.  Master or Above

9.  What is the monthly income in your family (NPR)

| a. 25K-35K |
| --- |
| b. 35K-45K |
| c. 45K-55K |
| d. Above 55K |
|  |

**Section B: Enabling factor related questions:**

 10.  Have you received any orientation/ training on COVID-19?

a.   Yes

b.  No

11.a. If yes, who provided training? ………………………..

 12.  How many hours do you work in a week?

a.   <= 40 hrs.

b.  >40 hrs.

13.  Do you get any extra allowances/incentives during COVID-19 outbreak?

a.   Yes

b.  No

14.a. If yes, what king of allowances? (multiple choices)

      i.      Daily Allowance

     ii.      Insurance

   iii.      Accommodation

   iv.      Travel/Fuel Allowance

    v.      Food Allowance

15.  How is the support and Co-ordination from palika?

a.   Poor

b.  Good

c.   Very Good

d.  Excellent

16.  Is supply of essential equipment and medicine enough at your health facility?

a.   Yes

b.  No

17.a. If not, what kind of equipment and medicines do you need? ……………………..

 18.  Is there any municipal level COVID-19 response committee/team in your palika?

a.   Yes

b.  No

19.  Is there any municipal COVID-19 response plan in your palika?

a.   Yes

b.  No

**Section C: Knowledge related questions**

20.  COVID-19 is :

a.   Virus

b.  Bacteria

c.   Fungus

d.  Parasite

21.  COVID-19 is transmitted by close contact with infected person?

a.   Yes

b.  No

22. The first case of novel coronavirus was identified in .....

A. Beijing

b. Shanghai

C. Wuhan, Hubei

D. Tianjin

 23.  In which age group the COVID-19 spreads?

a. COVID-19 occurs in all age groups.

b. Coronavirus infection is mild in children.

c. Older people and persons with pre-existing medical conditions are at high risk to develop serious illness.

d. All the above

 24.  Symptoms of COVID-19 are:

a.   Fever

b.  Cough

c.   Shortness of breath

d.  All the above

25.  The COVID-19 virus spreads via respiratory droplets of infected individuals.

a.   Yes

b.  No

26.  The isolation period for COVID-19 is:

a.   1 week

b.  2 weeks

c.   3 weeks

d.  More than 3 weeks

 27.  How to prevent from COVID-19?

a.   Clean hands with soap and water/ sanitizer

b.  Cover mouth and nose when coughing and sneezing

c.   Avoid close contact with those who show signs of flu

d.  Properly cook meats, eggs before eating

e.   All the above

 28.  COVID-19 vaccines are available in the market now

a.   Yes

b.  No

29.  Antibiotics are the first line treatment for COVID-19

a.   Yes

b.  No

30.  Health care workers are at high risk of COVID-19

a.   Yes

b.  No

31.  Full form of PPE:

a.   Personal Practical Equipment

b.  Personal Protective Equipment

c.   Personal Protection Equipment

d.  All the above

32. The Personal Protective Equipment includes:

a) Gloves

b) Gloves and apron

c) Gloves, apron, and face mask

d) Gloves, apron, face mask and eye protection

**Section D: Attitude related questions;**

 33.  Do you think you will get ill from COVID-19?

a.   Yes

b.  No

34.  If you get COVID-19, will you accept isolation in a Health facility?

a.   Yes

b.  No

35.  If COVID-19 vaccine was available, would you take it?

a.   Yes

b.  No

36.  Do you think wearing PPE reduces the chance of getting COVID-19?

a.   Yes

b.  No

37.  Do you think lockdown is the best way to prevent COVID-19?

a.   Yes

b.  No

38.  Do you feel bad when people don’t use masks while coming for treatment?

a.   Yes

b.  No

39.  Do you think COVID-19 will successfully be controlled?

a.   Yes

b.  No
